# Insights into the Acquisition of Virulence of Avian Influenza Viruses during a Single Passage in Ferrets

**DOI:** 10.3390/v11100915

**Published:** 2019-10-04

**Authors:** Jeffrey Butler, Deborah Middleton, Jessica Haining, Rachel Layton, Steven Rockman, Lorena E. Brown, Sandra Sapats

**Affiliations:** 1 The Commonwealth Scientific and Industrial Research Organisation, Australian Animal Health Laboratory (CSIRO-AAHL), Geelong 3219, Victoria, Australia; 2 Department of Microbiology and Immunology, The University of Melbourne at the Peter Doherty Institute of Infection and Immunity, Melbourne 3000, Victoria, Australia; 3 Seqirus, 63 Poplar Rd, Parkville 3052, Victoria, Australia

**Keywords:** avian influenza, ferret, mammalian adaptation, H5N1 influenza virus, systemic infection

## Abstract

Circulating avian influenza viruses pose a significant threat, with human infections occurring infrequently but with potentially severe consequences. To examine the dynamics and locale of the adaptation process of avian influenza viruses when introduced to a mammalian host, we infected ferrets with H5N1 viruses. As expected, all ferrets infected with the human H5N1 isolate A/Vietnam/1203/2004 showed severe disease and virus replication outside the respiratory tract in multiple organs including the brain. In contrast infection of ferrets with the avian H5N1 virus A/Chicken/Laos/Xaythiani-26/2006 showed a different collective pattern of infection; many ferrets developed and cleared a mild respiratory infection but a subset (25–50%), showed extended replication in the upper respiratory tract and developed infection in distal sites. Virus from these severely infected ferrets was commonly found in tissues that included liver and small intestine. In most instances the virus had acquired the common virulence substitution PB2 E627K but, in one case, a previously unidentified combination of two amino acid substitutions at PB2 S489P and NP V408I, which enhanced polymerase activity, was found. We noted that virus with high pathogenicity adaptations could be dominant in an extra-respiratory site without being equally represented in the nasal wash. Further ferret passage of these mutated viruses resulted in high pathogenicity in all ferrets. These findings illustrate the remarkable ability of avian influenza viruses that avoid clearance in the respiratory tract, to mutate towards a high pathogenicity phenotype during just a single passage in ferrets and also indicate a window of less than 5 days in which treatment may curtail systemic infection.

## 1. Introduction

Avian influenza A viruses are designated as either low pathogenicity avian influenza (LPAI) viruses or highly pathogenic avian influenza (HPAI) viruses based on their ability to cause severe disease and death in chickens in a laboratory setting. LPAI viruses are ubiquitous in wild birds throughout the world and HPAI viruses have evolved from subtype H5 and H7 LPAI viruses during replication in gallinaceous poultry. HPAI viruses have been reported in humans following direct or close contact with infected poultry, resulting in illness ranging from mild to severe. Mutations have been identified that appear to be necessary for avian influenza viruses to efficiently replicate in mammalian hosts, and it is now well documented that changes in the viral polymerase subunit PB2 (a key one being the E627K substitution [1]) are necessary for this. This change has been identified in many avian subtype viruses, including H5N1 and H7N9 [2,3,4,5,6,7]. Other amino acid (AA) changes such as PB2 D701N (detected less commonly in human isolates of H5N1 and H7N7 [8]), and the PB2 G590S, Q591R combination detected in A/H1N1pdm09 viruses [9], similarly facilitate the adaptation of influenza viruses of non-human origin to humans. The PB2 protein is part of the influenza virus polymerase complex (comprising the PB2, PB1, PA subunits). The role of the PB2 protein is to bind to the methylated cap structures attached to host cell pre-mRNA segments, which are then cleaved by the PA protein and used by the PB1 protein to initiate transcription of viral mRNA segments. Increased nuclear entry of the PB2 protein via enhanced binding interactions between PB2 proteins and different isoforms of the mammalian importin-α protein, have been identified as the molecular mechanisms which enable PB2 proteins incorporating AA changes such as E627K and D701N to adapt to mammalian hosts [10,11,12]. However, while many studies demonstrate the ability of avian influenza viruses to transition from low to high pathogenicity, these studies, which are most often performed in mice, usually involve serial passage in the mammalian host and do not provide information about when and how frequently the transition to the pathogenic phenotype occurs.

In order to understand the ability of avian influenza viruses to alter their pathogenic phenotype in a model better representing the human host, we infected naïve ferrets with an H5N1 avian influenza virus that initially demonstrated a low-pathogenic phenotype in ferrets. In addition to determining the mutations that gave rise to viruses with high pathogenicity for mammalian hosts, we investigated the rapidity and frequency of occurrence of such mutations and the location within the ferret organs where the mutated virus becomes established. These findings emphasize the potential of avian influenza viruses to select mutations that result in highly pathogenic infection, either in well documented virulence determinants or at additional sites, at disturbingly high frequency within a single passage in the ferret.

## 2. Materials and Methods

### 2.1. Cells and Viruses

African green monkey kidney (Vero) and Madin-Darby Canine Kidney (MDCK) cells were cultured in Eagle’s Minimal Essential Medium (EMEM, Thermo Fisher Scientific, Scoresby, Australia), supplemented with 10% (*v*/*v*) foetal calf serum (FCS, Thermo Fisher Scientific), 2 mM glutamine (Thermo Fisher Scientific), 10 mM *N*-2-hydroxyethylpiperazine-*N*′2-ethanesulfonic acid (HEPES, MP Biomedicals, Thermo Fisher Scientific), 100 units/mL penicillin (Sigma-Aldrich, Sydney, Australia), 100 µg/mL streptomycin (Sigma-Aldrich) and 2.5 µg/mL fungizone (Thermo Fisher Scientific). Human embryonic kidney-293T (293T) cells were cultured in Dulbecco’s Modified Eagle’s Medium (DMEM, Thermo Fisher Scientific), plus supplements. Two H5N1 influenza A viruses, the human isolate A/Vietnam/1203/2004 (A/Viet, phylogenetic clade 1) [13], and the avian virus A/Chicken/Laos/Xaythiani-26/2006 (A/Laos, clade 2.3) [14] were obtained from the influenza virus repository at the AAHL. Both viruses have a multibasic cleavage site enabling replication in extra-respiratory tract sites and are highly pathogenic in chickens. Reverse-engineered viruses, denoted with the prefix rg (such as rgA/Laos), were constructed from RNA extracted and cloned from the A/Laos and A/Viet viruses using the eight-plasmid system [15]. In preparation for inoculation into ferrets, all viruses were grown in the allantoic cavity of 9- to 11-day-old specific-pathogen-free embryonated hen’s eggs (Australian SPF services, Woodend, Australia) for up to 48 h. Harvested allantoic fluid was frozen at −80 °C and thawed immediately prior to use. The infectious titre of each stock was determined by titration in the allantoic cavity of embryonated eggs and the 50% egg infectious dose (EID_50_)/mL was calculated according to the method of Reed and Muench [16].

### 2.2. Ferret Experiments

Ferrets used in this study (sourced from IMVS, Adelaide, Australia) were 6–12 months old, of mixed gender, and all received food and water ad libitum while housed on a short light cycle (8 h light, 16 h dark), within the BSL-3 containment area at the AAHL. Prior to infection, a serum sample was taken from all ferrets and subjected to an influenza nucleoprotein (NP) competitive enzyme-linked immunosorbent assay (cELISA) capable of detecting antibodies against the influenza A virus nucleoprotein [17], to confirm the seronegative nature of all ferrets to influenza A viruses. Ferret body weights were recorded, and nasal washes sampled immediately prior to infection and at regular intervals post infection (PI). On day 0 of each experiment, all ferrets were anaesthetised by intramuscular injection of 20 mg/mL Ilium Xylazil-20 (Troy-Laboratories, Glendenning, Australia) then intranasally inoculated with 0.5 mL PBS containing 10^6^ EID_50_ of virus (inoculum split between the two nares). On the days indicated in each experiment, nasal washes were sampled by aspirating 0.5 mL of PBS into each nare and collecting the ejected fluid which was stored at −80 °C until further use. During virus inoculation and nasal washing, ferrets were anaesthetised with ketamine/medetomidine (50:50, 1 mg/kg), reversed with atipemazole. In order to sample tissues on the days indicated in each experiment, ferrets were anaesthetised with ketamine as above and subsequently euthanised via intra-cardiac injection of pentabarbitone (150 mg/kg). Tissue samples (approximately 100 mg) were taken in sterile 2 mL tubes containing approximately 100 mg of silica-carbide chips (Daintree Scientific, St. Helens, Australia) and either 1 mL of PBS plus antibiotics for subsequent virus titrations, or 700 µL RLT buffer for subsequent isolation of vRNA. All tissue samples were homogenised twice at maximum speed for 30 s using a FastPrep-24 Instrument (MP biomedicals, Thermo Fisher Scientific) prior to storage at −80 °C.

All experiments involving ferrets were conducted with the approval of the CSIRO-AAHL Animal Ethics Committee (permit number 1280, approved 15/09/08). All procedures were conducted according to the guidelines of the Australian Government National Health and Medical Research Council as described in the Australian code for the care and use of animals for scientific purposes [18].

### 2.3. Influenza NP cELISA

To detect broadly reactive influenza NP antibodies in ferret serum samples a competitive ELISA using plates coated with the NP from A/turkey/Ontario/6213/66 (H5N1) was used, as described by Selleck and Kirkland [17]. Sera that inhibited 60% of the monoclonal antibody binding in this assay were considered antibody positive, while those that inhibited ≤40% of the monoclonal antibody binding were considered antibody negative.

### 2.4. Titration of Infectious Virus on Vero Cells

To determine the infectious virus titre, samples were titrated on Vero cells. Confluent Vero monolayers grown in 75 cm^2^ flasks, were trypsinised and resuspended in 11 mL of EMEM plus supplements. For each 96-well plate, 1 mL of resuspended cells was added to 10 mL of EMEM plus supplements and 100 µL of this suspension loaded into each well of the plate. Nasal washes or 10% (*w*/*v*) tissue homogenates were serially diluted 10-fold in PBS and 100 µL aliquots were added to the 96-well plates freshly seeded with Vero cells. Plates were incubated at 37 °C, 5% (*v*/*v*) CO_2_ in a humidified incubator for five days. On the 5th day all wells were visually assessed by light microscope for the presence of cytopathic effect (absence of a monolayer accompanied by dead cells) and the 50% tissue culture infectious dose (TCID_50_) calculated according to the method of Reed and Muench [16].

### 2.5. Immunohistochemical Analysis of Viral Antigen

Following euthanasia, pieces of tissue ≤1 cm thick were fixed in neutral buffered formalin, embedded in paraffin and mounted on glass slides. Immunohistochemical staining of influenza viral antigen was performed using a rabbit polyclonal antiserum directed against a recombinant-expressed influenza viral NP (produced at the AAHL) and the DAKO EnVision^®^ + System-HRP-(AEC) (Agilent Technologies, Mulgrave, Australia), according to the manufacturer’s instructions. Each section was then lightly counterstained for 2 min in Mayer’s haematoxylin solution (Lillie’s modification) prior to examination.

### 2.6. RNA Isolation, RT-PCR and Sequencing

RNA was extracted from allantoic fluid, nasal washes and animal tissues using an RNeasy Minikit (QIAGEN, Chadstone, Australia) following the manufacturer’s instructions. RT-PCR was performed in a one-step reaction using SuperScript One-Step RT-PCR for Long Templates (Thermo Fisher Scientific) and relevant primers matching the sequence of each viral genome segment (sequences available upon request). DNA sequencing was performed at the AAHL DNA sequencing facility, using an ABI PRISM 377 automated DNA sequencer (Thermo Fisher Scientific). DNA sequences obtained were translated and the deduced AA sequences aligned using Clone Manager v9.11 (Scientific & Educational Software, USA).

### 2.7. PB2 627 Single Nucleotide Polymorphism Analysis

A semi-quantitative PCR single nucleotide polymorphism (SNP) assay was developed to allow rapid discrimination between the nucleotide codons encoding the AA residues E and K at PB2 627 of A/Laos. Viral RNA was subjected to reverse transcription to generate viral cDNA using the Quantitect Reverse Transcription Kit (QIAGEN) according to the manufacturer’s instructions. The cDNA templates were subjected to SNP analysis using a set of primers and probes (sequences available upon request) and a Type-it Fast SNP Probe PCR kit (QIAGEN), according to the manufacturer’s instructions. Reactions were performed on an AB7900HT Real time PCR system (Thermo Fisher Scientific), held for 5 min at 95 °C and then subjected to 45 cycles of PCR consisting of denaturing at 95 °C for 15 s and a combined annealing and extension at 60 °C for 30 s, using fast cycling parameters. Following the completion of each test, cycle threshold (Ct) values were assessed using the Sequence Detection System software version 2.3 (Thermo Fisher Scientific). Only those samples that produced a characteristic exponential amplification curve resulting in a *C*t value of ≤38 with either probe were considered to contain A/Laos virus encoding either PB2 627 E or K, or both.

### 2.8. Reverse-Engineered Viruses and Site-Directed Mutagenesis

All eight genome segments of the A/Laos and A/Viet H5N1 viruses were amplified by RT-PCR and incorporated into the pHW2000 reverse genetics (rg) virus rescue plasmid [15]. Reverse-engineered viruses were generated by transfection of all eight plasmids into a co-culture of 293T and MDCK cells, as previously described [15]. Supernatant that was haemagglutination positive was then amplified by a single virus passage in the allantoic cavity of 9- to 11-day-old embryonated hen’s eggs.

In order to investigate the effect of the PB2 627, 489 and NP 408 AA changes upon viral pathogenicity, plasmids encoding the A/Laos PB2 and NP as well as the A/Viet PB2 genome segments were subjected to site-directed mutagenesis using the GeneArt Site-Directed Mutagenesis System (Thermo Fisher Scientific) and relevant primer pairs (sequences available upon request). Following sequencing to confirm that the correct mutation had been achieved and that no other changes had been acquired, reverse-engineered viruses were generated by transfection of all eight plasmids into a co-culture of 293T and MDCK cells, as previously described [15]. The correct PB2 and/or NP sequence was confirmed by DNA sequencing for all generated viruses.

### 2.9. In Vitro Analysis of Viral Polymerase Activity

The in vitro replication efficiency of different viral polymerase combinations was determined using 293T cells transfected with 10 µg each of plasmids encoding NP, PB1, PB2, PA, in conjunction with 0.5 µL of the pRL-TK plasmid (Promega, Alexandria, Australia), which encodes the renilla luciferase internal control protein, and 10 µg of a plasmid encoding the firefly luciferase protein surrounded by the 5′ and 3′ non-coding regions of the A/Viet NP vRNA segment (pPOL-NP-Luc). The pPOL-NP-Luc plasmid was first constructed by PCR amplification of the firefly luciferase gene from the pGL3 promoter vector plasmid (Promega) using relevant primers engineered to encode either the A/Viet NP 5′ or 3′ non-coding regions, in frame with a 5′ or 3′ segment of the luciferase gene. The resulting PCR product was cloned into the pPOL-I plasmid, containing a truncated human RNA polymerase I promoter and the hepatitis delta virus ribozyme (kindly donated by Glenn Marsh, AAHL), to generate pPOL-NP-Luc. Each plasmid mixture was made up to 100 µL with Opti-MEM (Thermo Fisher Scientific); 1 µL of Lipofectamine 2000 (Thermo Fisher Scientific) was added and the mixture incubated at room temperature for 20 min. A confluent monolayer of 293T cells grown in a 75 cm^2^ flask, was trypsinised, pelleted and resuspended in 10 mL DMEM plus supplements. A 200 µL aliquot of this suspension was then added to each well of a white sterile flat-bottomed 24-well plate. Each 100 µL minigenome complex was then added to a single well and the plates incubated at 37 °C, 5% (*v*/*v*) CO_2_ in a humidified incubator for 5 h, after which another 500 µL of DMEM plus supplements was added to each well and the incubation continued overnight. The next day the relative activity of each polymerase complex was assessed using the Dual-Luciferase Reporter Assay System (Promega), and a Fluoroskan Ascent FL 96-well plate reader (Thermo Fisher Scientific). The polymerase activity in each sample was normalised by dividing the amount of firefly luciferase detected in each sample by the amount of renilla luciferase detected in each sample. The resulting level of luciferase expression in the negative control sample, containing only plasmids pRL-TK and pPOL-NP-Luc was set at one unit, and the polymerase activity detected in each sample expressed as a fold increase relative to this control. The relative luciferase activities of the different polymerase complexes were compared by a one-way analysis of variance test using GraphPad Prism 4 for windows (GraphPad Software, Inc., San Diego, CA, USA).

## 3. Results

### 3.1. A Single Passage of Avian H5N1 Virus in Ferrets Can Generate Virus Capable of Organ Dissemination

**Figure 1:**
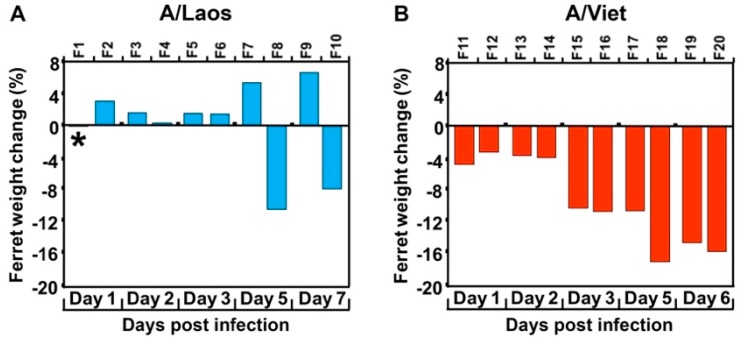
Weight loss induced by infection with avian and human H5N1 isolates. (**A**) Ferrets F1–F10 were infected with the avian H5N1 virus A/Laos (encoding PB2 627E) and (**B**) ferrets F11–F20 were infected with the human H5N1 isolate A/Viet (encoding PB2 627K). Ferrets were weighed immediately prior to virus inoculation on day 0 and again immediately prior to euthanasia on the indicated day of sampling. Values represent the percentage change from the initial ferret weights. * Indicates that a change in weight was not detected.

To understand the transition from low to high pathogenicity of H5N1 viruses in mammalian hosts, an avian H5N1 virus isolate (A/Laos) was inoculated into 10 ferrets (designated F1–10). For comparative purposes, a highly pathogenic human H5N1 virus isolate (A/Viet) was inoculated into an additional 10 ferrets (F11–20). Pairs of ferrets in each group were scheduled for sampling on days 1, 2, 3, 5 and 7 PI for assessment of body weight (Figure 1) and tissue sampling to detect the presence of infectious virus in multiple organs (Table 1). Weight loss and viral infection beyond the respiratory tract were observed in all ferrets inoculated with A/Viet from as early as day 1 PI and ferrets scheduled for sampling on day 7 had to be euthanised at the humane endpoint on day 6. In contrast, ferrets inoculated with A/Laos and sampled on days 1, 2 and 3 PI, had virus confined to the respiratory tract and showed body weights that either increased or showed no change (Figure 1, Table 1). By days 5 and 7 PI, one of each pair of ferrets sampled (F7 and F9) had completely cleared the virus. However, persistence of virus in nasal washes and systemic viral infection beyond the respiratory tract were accompanied by substantial weight losses in the remaining two A/Laos-inoculated ferrets, F8 and F10, which had been sampled on days 5 and 7 PI, respectively.

**Figure 2:**
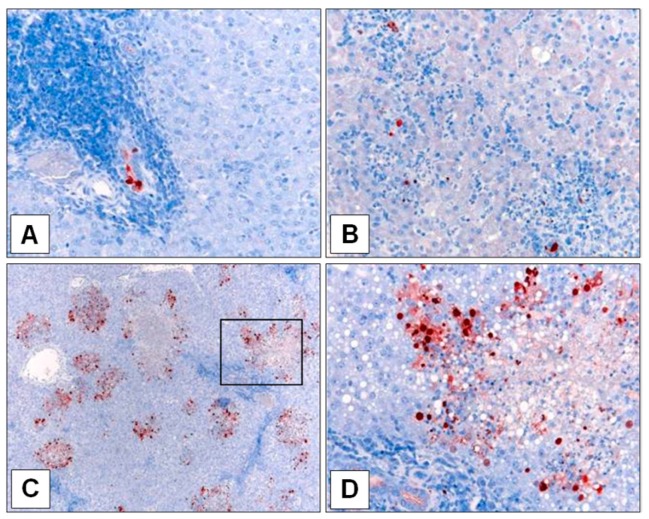
Immunostaining of H5N1 virus in liver samples of infected ferrets. Virus (red) was visualised in (**A**) liver of A/Laos-inoculated ferret F8 on day 5 PI.; (**B**) liver of A/Laos-inoculated ferret F10 on day 7 PI.; (**C**) and (**D**) liver of A/Viet-inoculated ferret F18 on day 5 PI. Boxed area in (**C**) denotes image shown in (**D**). Magnifications (**A**) 20×; (**B**) 20×; (**C**) 5×; (**D**) 20×.

Immunohistochemical staining of A/Laos-infected liver sections (Figure 2) revealed the presence of viral antigen confined to ductules in the liver of ferret F8, whereas viral antigen in the liver of ferret F10 appeared in more diffuse areas of the liver parenchyma, similar to that observed in the liver of ferrets inoculated with A/Viet. The A/Laos virus in the inoculum and the infected liver samples from ferrets F8 and F10 were fully sequenced to detect any AA differences (Table 2). Compared to the original inoculum, the virus recovered from the liver of ferret F8 showed three changes (PB2 I385V, PB2 N456D and PA E623G), whereas only a single AA change was detected in the A/Laos genome of the virus recovered from the liver of ferret F10 (PB2 E627K). Of these, only the PB2 E627K change led to an AA that corresponded to that in the equivalent position in A/Viet (Table 2).

To analyse whether any of these four AA substitutions had also occurred in other A/Laos-inoculated ferrets, segments of the genes encompassing the relevant coding regions were sequenced from all of the tissue and nasal wash samples from which A/Laos had been isolated (Table 1). The PB2 I385V, PB2 N456D and PA E623G changes were only observed in virus from the liver of ferret F8 (Table 3). Of interest, this ferret also had virus in the trachea that showed the PB2 E627K AA substitution and a mixed population in the nasal wash, indicative of potentially independent adaption pathways occurring in localised sites. PB2 E627K was also detected in virus from tissue samples of ferret F10 (liver, spleen and small intestine) but not the nasal wash. Virus samples from the remaining ferrets, where infection was confined to the respiratory tract, did not differ from the inoculum at these sites.

A SNP assay was used to further scrutinise the AA at PB2 627 in the A/Laos infected ferret tissues and nasal washes. The AA residues detected at PB2 627 by sequencing were confirmed by SNP assay in 10 of 14 samples, including the mixed population in the F8 nasal wash, with the remaining samples not yielding a result with either probe (Table 4). In summary the acquisition of PB2 627K by A/Laos replicating within ferrets F8 and F10 resulted in weight loss and an extra-respiratory infection in these ferrets, similar to that observed for ferrets infected with A/Viet, which encodes PB2 627K. Of interest, we noted that acquisition and/or selection of virus with PB2 627K in extra-respiratory tract tissues did not necessarily imply a dominance of this virus in the nasal wash at the site of shedding, as evidenced by the example of ferret F10.

### 3.2. Second Ferret Passage Confirms Pathogenicity Profile of the Mutated Viruses

To confirm whether the AA changes acquired by A/Laos during replication in ferrets F8 and F10 were responsible for the increase in pathogenicity and extra-respiratory replication, A/Laos virus recovered from the liver or nasal washes of ferrets F8 and F10 (referred to as A/F8/Liv, A/F8/NW, A/F10/Liv, A/F10/NW) were amplified in eggs and, in parallel with the parent A/Laos avian virus, were inoculated into groups of four ferrets, two of which were sampled on day 5 and the other two on day 7 PI (or earlier at the humane endpoint where required). Prior sequencing of the full coding region of the genome of these egg-amplified virus stocks revealed nine deduced AA differences from the A/Laos virus inoculum amongst the four ferret-passaged isolates (Table 5). In each case, the AA substitutions detected prior to virus amplification were maintained in the stock viruses, with those at PB2 627 being confirmed by SNP assay (Table A1). Additional differences from the parent virus were detected in PB1-F2 and NS1 of A/F10/Liv, PA of A/F8/NW, and PB2 and NP of A/F10/NW.

**Figure 3:**
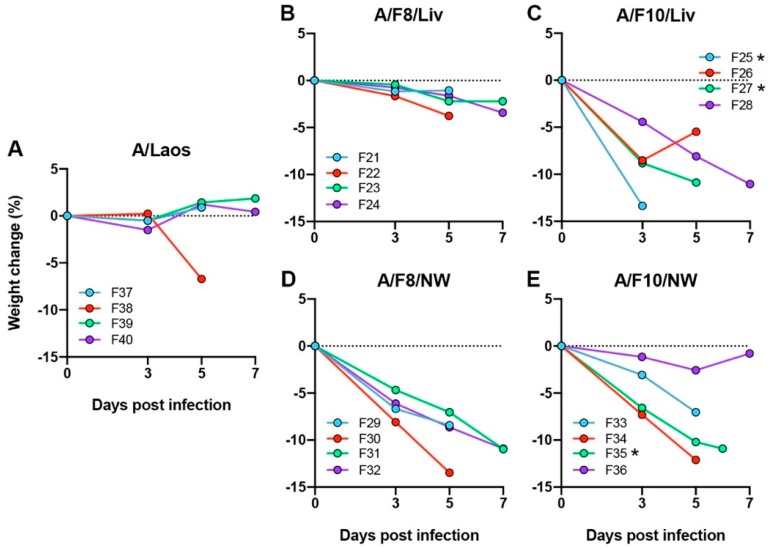
Weight changes following inoculation of ferrets with second passage H5N1 viruses. (**A**) A/Laos (PB2 627E); (**B**) A/F8/Liv (PB2 627E); (**C**) A/F10/Liv (PB2 627K); (**D**) A/F8/NW (PB2 627E/K); (**E**) A/F10/NW (PB2 627E)) H5N1 viruses. Two ferrets from each group were sampled on days 5 and 7 PI. * Ferrets were euthanised earlier than scheduled, at the humane endpoint.

As in the first experiment, the majority of ferrets (F37, F39 and F40) inoculated with the parent A/Laos avian isolate encoding PB2 627 E, experienced mild disease and maintained relatively stable body weights (Figure 3A). However, one of the four ferrets (F38) exhibited weight loss of 6.7% by day 5, developed an extra-respiratory tract infection involving the small intestine, liver and spleen (Table 6), and sequencing (Table 7) of those tissues that failed to yield a PB2 627 SNP assay result (Table A2) revealed that virus within this ferret had acquired the PB2 E627K AA substitution during this second virus passage. Virus could not be re-isolated from any of the tissues or nasal washes sampled from ferret F39, despite the development of NP-specific antibodies in the serum (serum samples taken from ferret F39 on days 0 and 7 PI, inhibited binding of the monoclonal antibody in the competition ELISA by 16 and 73% respectively), suggesting very rapid clearance of the inoculum in this ferret.

Ferrets inoculated with A/F10/NW (Figure 3E), an isolate that retained PB2 627E but had acquired PB2 S489P and NP V408I during the first virus passage, displayed a similar pattern of pathogenicity to that seen in ferrets inoculated with viruses that had acquired PB2 627K during the first virus passage. Three of the four ferrets inoculated with the A/F10/NW isolate displayed weight losses of 7.0–12.1%, and developed a systemic infection (Table 6), even though the virus replicating within two of these ferrets retained PB2 627E (Table 7 and Table A2). In comparison, the ferrets inoculated with the PB2 627K-encoding A/F10/Liv displayed weight losses of 5.5–13.4% (Figure 3C) and developed systemic viral infection (Table 6), and virus within these ferrets maintained PB2 627 K (Table 7 and Table A2). Similarly, ferrets inoculated with A/F8/NW encoding the PB2 627E/K mixed virus population developed weight losses of 8.4–13.5% (Figure 3D) and systemic infection (Table 6), while virus detected within these ferrets encoded exclusively PB2 627K (Table 7 and Table A2), highlighting the selection pressure for PB2 627K during H5N1 viral replication in ferrets.

Ferrets inoculated with the PB2 627E-encoding A/F8/Liv experienced mild disease with only minor weight losses of <4% (Figure 3B). However, a limited extra-respiratory infection of the small intestine was discovered in two of the ferrets infected with this virus (F21, F22) (Table 6), and in each of these ferrets the PB2 E627K AA substitution could be detected by SNP analysis or sequencing (Table 7 and Table A2), suggesting later mutation/selection of the highly pathogenic variant. Amongst all of the ferrets inoculated with the ferret-passaged H5N1 viruses, extra-respiratory infection and/or substantial weight loss were only observed in those ferrets where the infecting virus had acquired either the PB2 E627K single AA substitution, or the combined AA substitutions PB2 S489P and NP V408I.

### 3.3. Enhanced Pathogenicity of H5N1 in Ferrets Is Facilitated by the Combination of PB2 S489P or NP V408I, Not by Either PB2 S489P or NP V408I Individually

**Figure 4:**
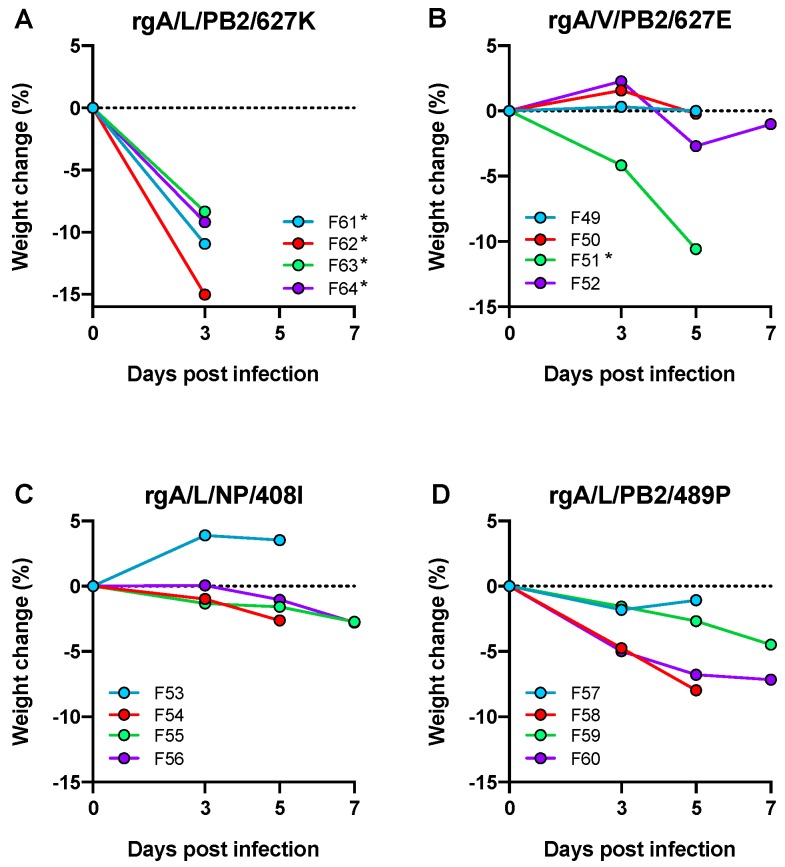
The effect of individual mutations at NP 408I or PB2 489P on pathogenicity. Percentage weight change following intranasal infection of ferrets with (**A**) rgA/L/PB2/627K; (**B**) rgA/V/PB2/627E; (**C**) rgA/L/NP/408I; or (**D**) rgA/L/PB2/489P viruses. * Ferrets were euthanised earlier than scheduled, at the humane endpoint.

To assess whether the increased pathogenicity observed in ferrets inoculated with A/F10/NW was due to the independent selection of viruses with individual adaptive mutations PB2 S489P or NP V408I, as opposed to a single population of virus with the two substitutions, the following reverse-engineered A/Laos viruses were generated and designated as follows: A/Laos encoding PB2 489P (rgA/L/PB2/489P) or NP 408I (rgA/L/NP/408I). A/Laos encoding PB2 627K (rgA/L/PB2/627K) and A/Viet encoding PB2 627E (rgA/V/PB2/627E) were also generated as controls. Each virus was inoculated into groups of four ferrets, two of which were scheduled for sampling on day 5 and the remainder on day 7 (Figure 4).

All four ferrets inoculated with rgA/L/PB2/627K required euthanasia prior to schedule due to reaching the humane end-point (Figure 4A). Each of these ferrets also developed a systemic infection (Table 8) and PB2 627K was retained by the virus within each of the infected tissues (Table A3 and Table A4). Three of the four ferrets inoculated with rgA/V/PB2/627E (Figure 4B) maintained a relatively stable body weight, although one ferret in this group (F51) required euthanasia on day 5 PI. Extra respiratory infection of the small intestine was observed in two ferrets from this group (F50 and F51) (Table 8), both of which acquired PB2 E627K AA substitutions (Table A3 and Table A4), in line with the frequency of 1–2 of four ferrets acquiring a high pathogenicity mutation within a single passage that we have consistently observed with the A/Laos viruses.

Amongst the ferrets inoculated with rgA/L/NP/408I, only minor weight losses of less than 3% were detected (Figure 4C) and extra-respiratory infection was only observed in the lymph node of ferret F53 (Table 8). However, PB2 E627K AA substitutions were detected in respiratory samples from two ferrets (F54 and F56) (Table A3 and Table A4), again similar to the pattern observed with introduction of non-adapted virus, with acquisition of a pathogenic genotype in 1–2 of four ferrets. Ferrets infected with rgA/L/PB2/489P (Figure 4D) showed a similar pattern with two of the four ferrets (F59, F60) developing extra-respiratory tract infection (Table 8), and the PB2 E627K AA substitution detected in one of these (F60) (Table A3). These data suggest that ferrets infected with rgA/L/PB2/489P and rgA/L/NP/408I do not experience the same collective pattern of disease as seen with a human-adapted input virus, such as the A/Viet virus encoding PB2 627K, where all ferrets show rapid weight loss and extra-respiratory virus spread, but rather the pattern characteristic of an avian-adapted input virus where a proportion of ferrets mutate to acquire virus of enhanced virulence. This would suggest that neither the PB2 S489P nor NP V408I AA substitution in isolation is sufficient to produce the high pathogenicity phenotype of human H5N1 isolates in the ferret model.

### 3.4. Polymerase Activity in Mammalian Cells Is Enhanced by Either PB2 627K or the Combination of PB2 S489P and NP V408I

**Figure 5:**
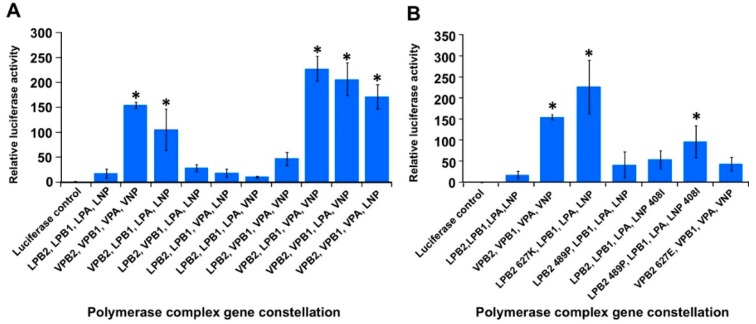
The effect of PB2 627K and the PB2 489P, NP 408I combination on viral polymerase activity in mammalian cells. Different combinations of (**A**) the pHWLaos and pHWViet PB2, PB1, PA and NP genome segment plasmids (designated as LPB2, VPB2, LPB1, VPB1, LPA, VPA, LNP and VNP, respectively); as well as (**B**) each of the plasmids encoding the single AA changes, pHWLaos PB2 489P (LPB2 489P), pHWLaos PB2 627K (LPB2 627K), pHWLaos NP 408I (LNP 408I) and pHWViet PB2 627E (VPB2 627E), were co-transfected into 293T cells along with plasmids pPOL-NP-LUC and pRL-TK. Following incubation at 37 °C for 24 h in a humidified incubator the relative luciferase activity of each polymerase complex was assessed using the Dual-Luciferase Reporter Assay System (Promega). Values (mean of three replicates ± standard error) are shown relative to the polymerase activity of the luciferase control (pRL-TK + pPOL-NP-LUC only) which represents one unit. Luciferase activity of each polymerase complex genetic constellation was compared to the luciferase activity of the native A/Laos polymerase constellation (LPB2, LPB1, LPA and LNP) using a one-way analysis of variance test. * *P* ≤ 0.05.

As H5N1 viruses encoding PB2 627K have been reported to demonstrate enhanced viral polymerase activity in mammalian cells [19,20], a dual luciferase reporter assay, capable of measuring viral polymerase activity, was used to assess whether the PB2 489 and NP 408 AA substitutions also alter viral polymerase activity. Different combinations of the A/Laos and A/Viet vRNP gene segment plasmids were co-transfected into 293T cells, and the relative expression of firefly and renilla luciferase was assessed 24 h later. As expected, the native A/Viet polymerase plasmid set (VPB2, VPB1, VPA and VNP) demonstrated significantly greater viral polymerase activity compared to the native A/Laos polymerase plasmid set (LPB2, LPB1, LPA and LNP) (Figure 5A). By alternating single genome segments within each viral polymerase set, it was revealed that every polymerase combination that included the A/Viet PB2 plasmid (VPB2) had enhanced viral polymerase activity compared to the viral polymerase combinations that included the A/Laos PB2 plasmid (LPB2) (Figure 5A). This observation demonstrated that the differences between the viral polymerase activities of A/Laos and A/Viet in mammalian cells are a result of AA differences in their PB2 genome segments. Subsequently six polymerase combinations were assessed using plasmids encoding single AA changes at position 627 of the A/Laos and A/Viet PB2 plasmids (LPB2 627K and VPB2 627E respectively) and at positions 489 and 408 of the A/Laos PB2 and NP plasmids (LPB2 489P and LNP 408I, respectively). In this way it was clearly observed that PB2 627K facilitated significantly increased polymerase activity, whereas the other introduced AA changes at PB2 S489P and NP V408I facilitated significantly increased polymerase activity only in combination, but not individually (Figure 5B).

## 4. Discussion

While similar studies of this type have been carried out previously [21,22], this study, performed in the ferret model, posed several important questions relating to the process of avian influenza adaption to the mammalian host that have yet to be fully considered in this model. We attempted to define how readily such adaption occurs, both in terms of frequency and rapidity. We also asked where in the animal do these viral adaptive mutations arise after intranasal infection and what are the implications of these mutations. Finally, we sought to understand the nature of the adapted virus.

Use of the A/Laos isolate that was considered to be of low pathogenicity in mammals [23], allowed for mammalian adaptation to be manifested by the detection of systemic spread by virtue of the multibasic cleavage site present in the HA of this virus. We showed that the pathogenic outcome was essentially binary; after infection of ferrets with the avian virus, the virus was either cleared from the respiratory tract by around 5 days, or the infection persisted in the upper respiratory tract beyond 5 days and became extra-respiratory. There were no cases of virus persisting beyond 5 days and not becoming extra-respiratory or of virus replicating in tissues outside the respiratory tract after being cleared from the respiratory tract. This is not to indicate that virus with adaptive mutations was not present in the animals until 5 days PI but that selection of such mutants and their rise to functional dominance took several days.

Another overarching observation was that the magnitude of virus loads in the nasal wash did not appear to predict systemic infection. Ferrets infected with A/Viet, all of which displayed systemic infection, had nasal wash titers ranging from 10^3.3^–10^6.8^, and those A/Laos-infected ferrets that ultimately showed viral replication outside the respiratory tract had nasal wash titers at both extremes of this range when systemic infection was detected. This is potentially related to the magnitude and effectiveness of innate defenses in individual animals. In terms of the frequency of adaptation, of the 20 ferrets sampled on days 5 or 7 PI with A/Laos virus or a virus inoculum (including reverse engineered virus) shown not to have adaptive mutations, eight of these (40%) acquired the capacity to grow in organs beyond the respiratory tract. Of these eight, all but one showed infectious virus in the small intestine and three showed infectious virus in the liver. Other sites were spleen, lymph node and olfactory bulb. Ferrets infected with virus already having adaptive mutations had a more extensive spread of infection starting from day 1 PI; of the 26 ferrets in this category (sampled from day 1–7 after infection with an adapted virus), the number of infected organs outside the respiratory tract ranged from one to seven (mean = 3.25). Those ferrets in which only one extra-respiratory organ was infected tended to be those sampled at the earlier time points (days 1–3) suggesting progressive seeding of virus throughout the animals with time. Interestingly, these early replication sites in ferrets infected with an already adapted virus, which were at disparate sites throughout the animals, did not correspond to the sites of first appearance of virus after infection with a non-adapted virus inoculum (small intestine, liver), perhaps suggesting that adaption is favoured in the small intestine or liver or that newly adapted virus more readily replicates in these sites.

When considering the implications of these findings for humans, as humans are infected with H5N1 viruses directly from an avian source and not currently from other infected humans, the majority will be expected to have a mild respiratory infection which is rapidly cleared and thus will probably go unreported. The unlucky and substantial percentage progressing to severe systemic disease are likely to experience a short initial lag phase in which viral replication may be inefficient before rapid expansion of the adapted virus in various organs, perhaps initially the small intestine or liver. Indeed, the incubation period in humans exposed to infected poultry has ranged from 2 to 10 days [24], and the mean length of illness for fatal H5N1 infections in humans is 9 to 10 days [25]. Furthermore, many reported cases of human H5N1 infection have been characterised by a period of illness apparently confined to either the gastrointestinal or respiratory tracts followed by a rapid systemic spread and subsequent death [26,27]. This clinical course of events reflects the observations made in ferrets during this study; failure to promptly clear the virus from the respiratory tract may provide an opportunity for the genetic changes that lead to a systemic infection to arise spontaneously due to errors in replication. This suggests that evidence of prolonged respiratory infection with H5N1 virus may serve as a useful prognostic indicator of systemic infection and highlights the need for prompt clearance of virus from the respiratory tract within the first 3 or 4 days in order to prevent the development of overwhelming systemic disease. Hence, the days immediately following a suspected infection likely represent a critical window of opportunity for the successful implementation of disease intervention strategies, such as the application of antiviral therapeutics, beyond which the challenge of successfully resolving a H5N1 infection presumably becomes increasingly difficult.

It should be noted that the viruses used in this study do not have the additional mutations in the HA identified by Imai et al. [28] and Herfst et al. [29] that are required for H5N1 viruses to become readily transmissible between ferrets by respiratory droplets, though it is understood that mutations in the polymerase genes that enhance viral replication in mammalian cells are an important step in this process [9,30,31,32,33]. The most widely studied of these polymerase mutations is PB2 E627K, which is commonly identified in H5N1 and H7N9 viruses isolated from humans [4,21,34]. The results of this study support the findings that the acquisition of the PB2 E627K mutation is the most common route to efficient replication in the mammalian host, though not the only one, and underscore the fact that such mutations can occur readily within a single passage in ferrets. The PB2 E627K mutation was initially described as being associated with overcoming cold-sensitivity [35], improving replication in the nasal turbinates and in tissue culture at 33 °C [35,36]. It was also suggested that this mutation provided a platform for further mutations that may facilitate human-to-human transmission. If this was the case, we might expect to see a dominance of virus bearing PB2 627K in the nasal wash of all ferrets in which systemic spread had occurred. While this was usually the case, ferret F10 was an example where the virus in the liver and other organs had acquired PB2 627K, however virus from this animal that remained in the nasal wash encoded PB2 627E. It is difficult to envisage that virus containing PB2 627K could be shed from such an animal when only PB2 627E could be isolated from the upper respiratory tract. This was not to say that the nasal wash virus from ferret F10 was unadapted. In fact, the amplified virus was shown to contain two mutations, PB2 S489P and NP V408I, which were, like the PB2 E627K mutation [37], able to enhance polymerase activity in mammalian cells when present in combination.

Although various combinations of mutations have been described in the literature for promoting mammalian adaptation [38,39,40,41], the PB2 S489P and NP V408I combination appears to be novel. The PB2 S489P single mutation has previously been described for its ability to enhance avian H5N1 polymerase activity in cultured human cells [42]. It has also been detected in combination with the PB2 E627K mutation during experimental infection of mice with an H7N1 highly pathogenic avian influenza virus that developed enhanced pathogenicity during murine infection [43]. Most combinations described to date involve the polymerase subunit genes [38,44,45], but an adaptive combination of PB2 and NP changes, has been previously described by Danzy et al. [46]. Interestingly, it was noted in their study that the NP mutation did not contribute to enhancing the polymerase activity which is contrary to our findings. During the course of our study we also identified other polymerase changes that occurred in the liver of ferret F8, namely PB2 I385V, PB2 N456D and PA E623G. However, the second ferret passage of virus from the F8 liver did not result in substantial weight loss and extra-respiratory tract replication was confined to the small intestine of two infected ferrets while the other two ferrets showed no systemic spread. It is tempting to suggest that these changes are in themselves insufficient to confer the pathogenic phenotype but may be intermediates for such pathogenic combinations to arise and reflect the dynamic nature of the influenza genome. The need for changes in both the PB2 and the NP for optimal replication efficiency are likely explained by the requirement of these proteins to acquire specificity for importin-α7 in mammalian cells as opposed to importin-α3, which avian viruses depend on for entry of the genome into the nucleus for replication [12], or additionally for other barriers that have been described [47] to be overcome. Perhaps the small intestine and liver, where we observe the adaptive process initially manifesting, provide sufficient levels of replication to increase the probability of a second mutation to occur.

The work presented in this study, using the ferret model, demonstrates that H5N1 viruses are capable of mutating to achieve high levels of replication in mammals by acquiring polymerase changes such as PB2 E627K or the PB2 S489P, NP V408I combination, rapidly and at high frequency. Furthermore, this process effectively converts an infection which is initially of low pathogenicity in the newly infected mammalian host to one of high pathogenicity in the course of a single virus passage in less than 5 days. These findings highlight the risks posed by ongoing human H5N1 infections where such mutations may form the foundation for further mutation towards a transmissible phenotype and reinforce the importance of preventing such infections.

## Figures and Tables

**Table 1:** Human-adapted H5N1 virus produces a more rapid systemic infection in ferrets than avian H5N1 virus.

	A/Laos										A/Viet									
	Day 1 ^a^		Day 2		Day 3		Day 5		Day 7		Day 1		Day 2		Day 3		Day 5		Day 6	
Sample	F1 ^b^	F2	F3	F4	F5	F6	F7	F8	F9	F10	F11	F12	F13	F14	F15	F16	F17	F18	F19	F20
Nasal wash	3.8 ^c^	6.8	4.6	3.0	3.3	<	<	6.0	<	3.8	6.8	3.3	5.5	4.6	5.6	5.3	5.3	4.3	5.0	3.8
Lung	<	<	<	<	<	4.0	<	<	<	<	5.6	4.8	<	4.1	4.3	<	<	4.8	<	6.8
Trachea	<	<	<	<	<	<	<	3.8	<	<	3.5	3.6	<	<	<	<	3.0	6.1	<	3.9
Small intestine	<	<	<	<	<	<	<	4.6	<	3.6	<	<	<	<	<	2.9	3.3	5.5	4.1	4.6
Large intestine	<	<	<	<	<	<	<	<	<	<	<	<	<	<	<	<	<	<	<	<
Liver	<	<	<	<	<	<	<	6.3	<	4.6	<	<	<	4.5	<	<	3.8	5.8	6.3	6.0
Pancreas	<	<	<	<	<	<	<	<	<	<	<	<	<	<	<	<	<	<	<	<
Heart	<	<	<	<	<	<	<	<	<	<	<	4.0	<	<	4.3	<	3.0	4.5	<	5.0
Spleen	<	<	<	<	<	<	<	<	<	7.0	<	<	5.0	<	5.8	5.8	3.8	6.8	4.8	<
Kidney	<	<	<	<	<	<	<	<	<	<	<	<	<	<	<	<	<	6.8	<	<
Thymus	<	<	<	<	<	<	<	<	<	<	<	<	5.0	<	3.8	4.8	<	7.0	<	5.8
Hindbrain	<	<	<	<	<	<	<	<	<	<	<	<	<	<	<	6.0	<	4.5	<	8.3
Lymph node	<	<	<	<	<	<	<	<	<	<	3.3	<	3.0	<	3.0	<	<	<	<	<

^a^ Day post infection (PI) of sampling of ferret tissues; ^b^ identification number of ferrets sampled on specified day; ^c^ samples in PBS with antibiotics representing 1 mL nasal wash or 10% (*w*/*v*) tissue homogenate were titrated on Vero cells to determine TCID_50_/mL. < Indicates no virus detected. The lower limit of detection was ≤2.8 log_10_ TCID_50_/mL for nasal wash, trachea, retropharyngeal lymph node, heart, spleen, small intestine and thymus, and ≤3.8 log_10_ TCID_50_/mL for the other tissue samples.

**Table 2:** A/Laos amino acid (AA) sequence changes in ferret liver samples.

		A/Laos			A/Viet
		Inoculum	Liver Sample		Inoculum
Protein	AA Position ^a^		F8 ^b^	F10	
PB2	385	I ^c^	V	I	I
	456	N	D	N	N
	627	E	E	K	K
PA	623	E	G	E	E

^a^ Based on deduced AA sequence of A/Viet proteins (GenBank accession numbers: AAW80708 and AAW80714); ^b^ ferret identification number; ^c^ the AA present at each position (single letter AA code).

**Table 3:** A/Laos AA sequence changes in tissues and nasal washes.

Day PI	Ferret	Sample	PB2 385 ^a^	PB2 456	PB2 627	PA 623
		A/Laos inoculum	I	N	E	E
1	F1	Nasal wash	I	N	E	E
	F2	Nasal wash	I	N	E	E
2	F3	Nasal wash	I	N	E	E
	F4	Nasal wash	I	N	E	E
3	F5	Nasal wash	I	N	E	E
	F6	Lung	I	N	E	E
5	F8	Nasal wash	I	N	E/K ^b^	E
		Trachea	I	N	K	E
		Liver	V	D	E	G
		Small intestine	I	N	E	E
7	F10	Nasal wash	I	N	E	E
		Liver	I	N	K	E
		Spleen	I	N	K	E
		Small intestine	I	N	K	E

^a^ Viral protein and AA number; ^b^ mixed population within sample.

**Table 4:** A/Laos PB2 627 single nucleotide polymorphism (SNP) analysis following infection of ferrets.

			Ct Values ^a^	
Day PI	Ferret ^b^	Sample	PB2 627E	PB2 627K
		A/Laos inoculum	19.9	- ^c^
1	F1	Nasal wash	36.1	-
	F2	Nasal wash	27.0	-
2	F3	Nasal wash	30.9	-
	F4	Nasal wash	31.9	-
3	F5	Nasal wash	34.2	-
	F6	Lung	27.1	-
5	F8	Nasal wash	31.8	31.8
		Trachea	-	-
		Liver	31.2	-
		Small intestine	-	-
7	F10	Nasal wash	-	-
		Liver	-	23.6
		Spleen	-	30.1
		Small intestine	-	-

^a^ Mean cycle threshold (Ct) values for the PB2-627E-FAM and PB2-627K-VIC probes, from duplicate non-standardised Q-PCR reactions; ^b^ ferret identification number; ^c^ indicates that a cycle threshold (Ct) value of ≤38 was not obtained from this probe and sample combination.

**Table 5:** Amino acid sequence changes in ferret-passaged A/Laos virus egg-amplified stocks.

Virus	PB2 385 ^a^	PB2 456	PB2 489	PB2 627	PB1-F2 4	PA 623	PA 330	NP 408	NS1 175
A/Laos	I ^b^	N	S	E	G	E	I	V	V
A/F8/Liv	V	D	S	E	G	G	I	V	V
A/F10/Liv	I	N	S	K	E	E	I	V	I
A/F8/NW	I	N	S	E/K ^c^	G	E	V	V	V
A/F10/NW	I	N	P	E	G	E	I	I	V

^a^ Numbering as in Table 2; ^b^ the AA present at each position as determined from sequence analysis of duplicate RT-PCR reactions; ^c^ mixed population within sample.

**Table 6:** Ferret-passaged H5N1 isolates encoding PB2 627K produce a more widespread systemic infection upon a second virus passage in ferrets.

	A/Laos				A/F8/Liv				A/F10/Liv				A/F8/NW				A/F10/NW			
**Sample**	**F37** ^**a**^	**F38**	**F39**	**F40**	**F21**	**F22**	**F23**	**F24**	**F25**	**F26**	**F27**	**F28**	**F29**	**F30**	**F31**	**F32**	**F33**	**F34**	**F35**	**F36**
Nasal wash Day 3	4.5 ^b^	3.6	< ^c^	3.8	<	5.6	<	3.6	5.8	3.8	5.8	4.3	3.8	2.3	<	4.8	3.8	4.6	3.8	5.0
Nasal wash Day 5	3.0	6.3	<	4.0	2.5	5.8	3.8	4.1	- ^d^	4.8	5.3	5.5	4.0	2.8	<	3.6	4.8	4.0	4.6	4.3
Nasal wash Day 7			<	<			<	<				4.5			<	2.8			-	2.3
	**Day 5**		**Day 7**		**Day 5**		**Day 7**		**Day 3**	**Day 5**		**Day 7**	**Day 5**		**Day 7**		**Day 5**		**Day 6**	**Day 7**
Lung ^e^	7.0 ^e^	3.3	<	<	6.1	5.3	<	<	6.0	6.1	3.6	<	4.1	3.5	<	5.8	4.3	4.8	5.8	<
Trachea	<	<	<	<	<	4.0	<	<	6.0	5.6	5.6	<	4.3	4.3	3.3	4.3	<	4.3	4.8	<
Small intestine	<	4.3	<	<	5.0	3.8	<	<	<	<	4.5	<	5.0	5.8	4.0	<	3.3	4.8	4.8	<
Liver	<	5.0	<	<	<	<	<	<	5.5	5.3	5.6	4.6	<	5.8	6.5	4.8	<	<	7.3	<
Pancreas	<	<	<	<	<	<	<	<	<	<	<	<	<	<	<	<	<	<	5.8	<
Heart	<	<	<	<	<	<	<	<	3.6	<	3.3	<	<	<	<	<	<	<	<	<
Spleen	<	4.3	<	<	<	<	<	<	5.3	<	4.8	<	<	3.3	<	<	3.8	3.5	<	<
Kidney	<	<	<	<	<	<	<	<	<	<	<	<	<	<	<	<	<	<	<	<
Thymus	<	<	<	<	<	<	<	<	5.3	5.8	4.8	<	5.3	3.3	<	<	<	3.8	4.3	<
Hindbrain	<	<	<	<	<	<	<	<	4.1	5.3	6.0	<	<	7.0	<	4.6	<	7.5	<	<
Olfactory bulb	<	<	<	<	<	<	<	<	<	3.8	<	4.8	<	4.3	<	5.1	<	<	5.8	<
Lymph node	<	<	<	<	<	<	<	<	<	<	<	<	<	<	<	<	<	<	<	<

^a^ Identification number of ferrets sampled on specified day; ^b^ values are individual virus titres from 1 mL nasal wash samples collected in PBS with antibiotics and titrated on Vero cells. The lower limit of virus detection in nasal washes was ≤2.8 log_10_ TCID_50_/mL; ^c^ < indicates that virus was absent or below the lower limit of detection in the collected sample; ^d^ indicates no sample, ferret euthanised prior to this sampling day; ^e^ values are individual virus titres from 10% (*w*/*v*) tissue homogenates, collected and homogenised in sterile PBS with antibiotics, and titrated on Vero cells. The lower limit of virus detection was ≤2.8 log_10_ TCID_50_/mL for the lung, trachea, retropharyngeal lymph node, heart, spleen, small intestine and thymus samples, and ≤3.8 log_10_ TCID_50_/mL for the other tissue samples.

**Table 7:** Identification by sequencing of the AA at PB2 627 in A/Laos following a second virus passage in ferrets.

Virus	Day PI	Ferret ^a^	Sample	PB2 627 ^b^
A/F8/Liv	5	F21	Small intestine	K ^c^
		F22	Small intestine	- ^d^
A/F10/Liv	3	F25	Hindbrain	K
	5	F26	Liver	K
		F27	Liver	-
			Heart	-
A/F8/NW	7	F31	Trachea	-
			Small intestine	-
			Liver	-
A/F10/NW	5	F33	Small intestine	-
			Spleen	-
		F34	Trachea	E
			Small intestine	E
			Spleen	-
			Thymus	E
	6	F35	Small intestine	E
			Thymus	E
A/Laos	5	F38	Lung	K
			Small intestine	K
			Liver	K
			Spleen	K

^a^ Ferret identification number; ^b^ numbering as in Table 2; ^c^ the presence of E or K at PB2 627; ^d^ indicates that an RT-PCR product could not be obtained from this tissue sample.

**Table 8:** Reverse-engineered viruses with mutations at either NP 408I or PB2 489P did not exhibit a high pathogenicity phenotype.

	rgA/V/PB2/627E				rgA/L/NP/408I				rgA/L/PB2/489P				rgA/L/PB2/627K			
**Sample**	**F49** ^**a**^	**F50**	**F51**	**F52**	**F53**	**F54**	**F55**	**F56**	**F57**	**F58**	**F59**	**F60**	**F61**	**F62**	**F63**	**F64**
Nasal wash Day 3	2.8 ^b^	2.8	4.6	2.8	2.8	4.3	4.9	4.6	4.6	4.8	4.1	3.8	7.6	5.0	4.1	6.0
Nasal wash Day 5	< ^c^	<	7.8	<	2.9	5.8	5.0	6.3	5.0	4.6	4.6	5.5	- ^d^	-	-	-
Nasal wash Day 7	-	-	-	<	-	-	<	<	-	-	3.6	4.6	-	-	-	-
	**Day 5**			**Day 7**	**Day 5**		**Day 7**		**Day 5**		**Day 7**		**Day 3**		**Day 4**	
Lung	5.3 ^e^	<	5.6	<	5.0	5.6	<	<	3.6	5.3	4.8	<	<	4.3	5.1	6.8
Trachea	<	4.3	<	<	<	3.3	<	<	4.3	3.8	<	3.0	4.8	4.8	6.0	5.6
Small intestine	<	3.0	5.5	<	<	<	<	<	<	<	4.0	4.8	<	<	<	5.5
Liver	<	<	<	<	<	<	<	<	<	<	<	<	<	4.6	4.8	<
Pancreas	<	<	<	<	<	<	<	<	<	<	<	<	<	3.8	<	<
Heart	<	<	<	<	<	<	<	<	<	<	<	<	<	3.8	3.0	3.0
Spleen	<	<	<	<	<	<	<	<	<	<	<	<	4.0	<	4.6	4.3
Kidney	<	<	<	<	<	<	<	<	<	<	<	<	<	<	<	<
Thymus	<	<	<	<	<	<	<	<	<	<	<	<	<	3.8	4.8	6.0
Hindbrain	<	<	<	<	<	<	<	<	<	<	<	<	<	<	<	6.6
Olfactory bulb	<	<	<	<	<	<	<	<	<	<	<	4.6	<	<	<	<
Lymph node	<	3.0	<	<	3.1	<	<	<	<	<	<	<	<	6.0	4.1	3.8

^a^ Identification number of ferrets euthanised on each sampling day; ^b^ values are individual virus titres from 1 mL nasal wash samples collected in PBS with antibiotics and titrated on Vero cells. The lower limit of virus detection in nasal washes was ≤2.8 log_10_ TCID_50_/mL; ^c^ < indicates that virus was absent or below the lower limit of detection in the collected sample; ^d^ indicates no sample, ferret euthanised prior to this sampling day; ^e^ values are individual virus titres from 10% (*w*/*v*) tissue homogenates, collected and homogenised in sterile PBS with antibiotics, and titrated on Vero cells. The lower limit of virus detection was ≤2.8 log_10_ TCID_50_/mL for the lung, trachea, retropharyngeal lymph node, heart, spleen, small intestine and thymus samples, and ≤3.8 log_10_ TCID_50_/mL for the other tissue samples.

## GenBank

A/Laos and A/Viet genome sequences cloned during the course of this project are available in GenBank under accession numbers HM006748 to HM006763.

## Appendix A

**Table A1:** PB2 627 SNP analysis of A/Laos ferret-passaged isolates following amplification in eggs.

	Ct Values ^a^	
Isolated Virus	PB2 627E	PB2 627K
A/F8/Liv	19.9	- ^b^
A/F10/Liv	-	21.1
A/F8/NW	22.2	23.2
A/F10/NW	22.3	-

^a^ Mean cycle threshold (Ct) values for the PB2-627E-FAM and PB2-627K-VIC probes, from duplicate non-standardised Q-PCR reactions; ^b^ indicates that a Ct value of ≤38 was not obtained from this probe and sample combination.

**Table A2:** SNP analysis of the AA at PB2 627 in A/Laos following the second virus passage in ferrets.

				Ct Values ^a^	
Virus	Day PI	Ferret ^b^	Sample	PB2 627E	PB2 627K
A/F8/Liv	5	F21	Lung	35.0	- ^c^
			Small intestine	-	-
		F22	Lung	35.9	31.6
			Trachea	36.5	-
			Small intestine	-	-
A/F10/Liv	3	F25	Lung	-	28.7
			Trachea	-	28.4
			Liver	-	31.0
			Heart	-	37.5 ^d^
			Spleen	-	34.8
			Thymus	-	29.9
			Hindbrain	-	-
	5	F26	Lung	-	29.8
			Trachea	-	30.6
			Liver	-	-
			Thymus	-	27.2
			Hindbrain	-	27.3
			Olfactory bulb	-	27.2
		F27	Lung	-	34.4
			Trachea	-	30.8
			Small intestine	-	36.9 ^d^
			Liver	-	-
			Heart	-	-
			Spleen	-	37.4 ^d^
			Thymus	-	31.8
			Hindbrain	-	25.9
	7	F28	Liver	-	35.5
			Olfactory bulb	-	27.6
A/F8/NW	5	F29	Lung	-	37.7 ^d^
			Trachea	-	37.4
			Small intestine	-	34.2
			Thymus	-	31.4
		F30	Lung	-	32.6
			Trachea	-	34.8
			Small intestine	-	37.2
			Liver	-	33.6
			Spleen	-	36.5 ^d^
			Thymus	-	37.4 ^d^
			Hindbrain	-	32.5
			Olfactory bulb	-	34.7
	7	F31	Trachea	-	-
			Small intestine	-	-
			Liver	-	-
		F32	Lung	-	37.6
			Trachea	-	33.9
			Liver	-	31.8
			Hindbrain	-	31.0
			Olfactory bulb	-	36.6 ^d^
A/F10/NW	5	F33	Lung	34.9	-
			Small intestine	-	-
			Spleen	-	-
		F34	Lung	34.5	-
			Trachea	-	-
			Small intestine	-	-
			Spleen	-	-
			Thymus	-	-
			Hindbrain	37.7	-
	6	F35	Lung	33.6	-
			Trachea	35.9	-
			Small intestine	-	-
			Liver	29.3	-
			Pancreas	32.3	-
			Thymus	-	-
			Olfactory bulb	29.8	-
A/Laos	5	F37	Lung	31.8	-
		F38	Lung	-	-
			Small intestine	-	-
			Liver	-	-
			Spleen	-	-

^a^ Mean cycle threshold (Ct) values for the PB2-627E-FAM and PB2-627K-VIC probes, from duplicate non-standardised Q-PCR reactions; ^b^ ferret identification number; ^c^ indicates that a Ct value of ≤38 was not obtained for this probe and sample combination; ^d^ a Ct value was only detected in one of the duplicate reactions.

**Table A3:** A/Laos PB2 627 SNP analysis following infection of ferrets with reverse-engineered H5N1 viruses.

				Ct Values ^a^	
Virus	Day PI	Ferret ^b^	Sample	PB2 627E	PB2 627K
rgA/L/NP/408I	5	F53	Lung	26.4	- ^c^
			Lymph node	-	-
		F54	Lung	-	24.4
			Trachea	30.5	-
rgA/L/PB2/489P	5	F57	Lung	27.4	-
			Trachea	25.9	-
		F58	Lung	22.2	-
			Trachea	30.7	-
	7	F59	Lung	28.5	-
			Small intestine	-	-
		F60	Trachea	-	33.3
			Small intestine	-	-
			Olfactory bulb	29.4	-
rgA/L/PB2/627K	3	F61	Trachea	-	28.7
			Spleen	-	32.2
		F62	Lung	-	28.6
			Trachea	-	29.9
			Liver	-	31.0
			Pancreas	-	-
			Heart	-	33.8
			Thymus	-	28.2
			Lymph node	-	22.9
	4	F63	Lung	-	30.3
			Trachea	-	25.6
			Liver	-	-
			Heart	-	34.8
			Spleen	-	28.5
			Thymus	-	29.5
			Lymph node	-	27.2
		F64	Lung	-	31.5
			Trachea	-	26.2
			Small intestine	-	-
			Heart	-	36.3
			Spleen	-	33.2
			Thymus	-	32.6
			Hindbrain	-	31.4
			Lymph node	-	30.1

^a^ Mean cycle threshold (Ct) values for the PB2-627E-FAM and PB2-627K-VIC probes, from duplicate non-standardised Q-PCR reactions; ^b^ ferret identification number; ^c^ indicates that a Ct value of ≤38 was not obtained for this probe and sample combination.

**Table A4:** PB2 627 sequencing following infection of ferrets with rg H5N1 viruses.

Virus	Day PI	Ferret ^a^	Sample	PB2 627 ^b^
rgA/V/PB2/627E	3	F49	Nasal wash	- ^c^
	5	F49	Lung	E ^d^
	3	F50	Nasal wash	-
	5	F50	Trachea	E/K ^e^
		F50	Small intestine	-
		F50	Lymph node	K
	3	F51	Nasal wash	E
	5	F51	Nasal wash	E/K
		F51	Lung	K
		F51	Small intestine	-
	3	F52	Nasal wash	E
rgA/L/NP/408I	3	F53	Nasal wash	-
	5	F53	Nasal wash	-
	3	F54	Nasal wash	E
	5	F54	Nasal wash	E
	3	F55	Nasal wash	E
	5	F55	Nasal wash	E
	3	F56	Nasal wash	E
	5	F56	Nasal wash	E/K
rgA/L/PB2/489P	3	F57	Nasal wash	E
	5	F57	Nasal wash	E
	3	F58	Nasal wash	E
	5	F58	Nasal wash	E
	3	F59	Nasal wash	E
	5	F59	Nasal wash	E
	7	F59	Nasal wash	E
	3	F60	Nasal wash	E
	5	F60	Nasal wash	E
	7	F60	Nasal wash	E
rgA/L/PB2/627K	3	F61	Nasal wash	K
		F62	Nasal wash	K
		F63	Nasal wash	K
		F64	Nasal wash	K

^a^ Ferret identification number; ^b^ numbering as in Table 2; ^c^ indicates that an RT-PCR product could not be obtained from this tissue sample; ^d^ the presence of E or K at PB2 627; ^e^ mixed population within sample.
